# Management of a measles outbreak in a reception facility for asylum seekers in Regensburg, Germany

**DOI:** 10.3205/dgkh000322

**Published:** 2019-06-03

**Authors:** Benedikt M. J. Lampl, Markus Lang, Matthias Pregler, Marc Zowe, Rainer Beck, Katharina Schönberger

**Affiliations:** 1 Public Health Department Regensburg, Landratsamt Regensburg, Germany; 2 District Government Oberpfalz, Bavaria, Germany; 3 Private Practice, Regensburg, Germany; 4 Bavarian Health and Food Safety Authority (LGL), Germany

**Keywords:** measles outbreak, asylum seekers, reception facility, infection control

## Abstract

In July/August 2018, a measles outbreak occurred in a reception facility for asylum seekers in Regensburg, Bavaria, Germany. A five-year-old child and an 18-year-old man from Moldova were affected. At the time of the report, 491 people were accommodated at the facility. The outbreak was limited to the two cases mentioned by the consistent use of infection control measures. Decisive factors for successfully combating this outbreak were, in particular, the close cooperation of the local public health authority (Gesundheitsamt) with the district government officials, the institution’s management, and the general practitioners on site. The measures taken included the early information of all parties involved, the timely and repeated implementation of containment vaccinations, a consequent segregation of contagion and/or disease suspects and the critical consideration of each individual case in connection with the separate risk-adapted segregation of particularly vulnerable cohorts.

## Zusammenfassung

Im Juli/August 2018 kam es in einer Massenunterkunft für Asylsuchende in Regensburg zu einem Masernausbruch. Betroffen waren ein fünfjähriges Kind und ein 18-jähriger Mann aus Moldawien. Zum Zeitpunkt der Meldung waren 491 Personen in der Unterkunft untergebracht. Der Ausbruch konnte durch den konsequenten Einsatz von Infektionsschutzmaßnahmen auf die genannten beiden Fälle limitiert werden. Maßgeblich für die erfolgreiche Bekämpfung dieses Ausbruchs waren insbesondere die enge Zusammenarbeit des Gesundheitsamtes mit den Verantwortlichen der Regierung, der Einrichtungsleitung und den vor Ort tätigen Allgemeinmedizinern. Die ergriffenen Maßnahmen beinhalteten die frühzeitige Information aller Beteiligten, die rechtzeitige und wiederholte Durchführung von Riegelungsimpfungen, eine konsequente Absonderung von Ansteckungs- und/oder Krankheitsverdächtigen sowie die kritische Abwägung jedes Einzelfalles im Zusammenhang mit der getrennten risikoadaptierten Absonderung von besonders gefährdeten Kohorten.

## Background

Measles is an infectious disease with a high contagion index, but is preventable by vaccination [1]. Although measles is commonly regarded as a classic childhood disease and often considered harmless, it can lead to serious complications, such as acute encephalitis (cumulative incidence 1:1000 patients) or subacute sclerosing panencephalitis (SSPE, cumulative incidence 4–11:100,000 cases of measles with a significantly higher risk for children under five years of age), with possibly the most severe neurological sequelae and death of the person affected [1]. Measles cases and outbreaks in group housing pose a particular challenge for infection control, as a large number of contagion suspects and particularly vulnerable people (pregnant women and infants) live together in a confined space, and examinations and implementation of measures require great effort [2], [3], [4], [5].

### Outbreak detection

On 25/07/2018, a suspected case of measles in a reception facility for asylum seekers was reported to the Regensburg public health department (Gesundheitsamt). The suspected patient was a five-year-old male Moldavan child. As far as could be determined, he had not been vaccinated against measles. Clinically, a typical exanthema as well as fever, catarrhal symptoms, and conjunctivitis were found. The disease was confirmed by the laboratory on 26/07/2018 (measles-specific IgM posi-tive). Immediately upon receipt of the laboratory confir-mation, the local public health department provided the head of the facility with of a plan of action (see Table 1 (Tab. 1)). The index patient was isolated with his family. On 27/07/2018 the government of the district (Regierung der Oberpfalz) was informed about the event as well as the local children’s hospital. At the time of first notification, 491 people were accommodated at the facility. The investigation also revealed that a total of 41 asylum seekers had been transferred to a neighboring facility during the ten-day period prior to the rash manifestation of the index patient. The public health department responsible was informed on 27/07/2018 about the transfers.

## Methods

Case definitions were applied according to the Robert Koch Institute (RKI) [6]. The clinical presentation of measles is defined as a generalized (maculopapular) rash and fever as well as cough, catarrh, and/or conjunctivitis. For laboratory diagnostics, the following criteria apply: direct detection of pathogens: antigen detection (e.g. IFT, immuno-colorimetric test), pathogen isolation (cultural), detection of nucleic acids (e.g. PCR); or indirect [serological] detection: IgM antibody detection (e.g. ELISA, IFT) and IgG antibody detection (marked change between two samples; e.g. ELISA, IFT, NT). A genotype analysis to identify transmission chains could not be achieved in the Regensburg cases. For characteristics of the cases, see Table 2 (Tab. 2).

### Outbreak control measures

Immediately following the first laboratory confirmation, the residents of the facility were informed about the disease and the opportunity of vaccination through an information session and notices posted in the facility. The general practitioner on site and his team performed a first set of containment vaccinations. However, the response from the asylum-seekers was rather low. Accurate identification of contagion suspects was not possible under the given circumstances in group housing, and consistent segregation within the facility was difficult because contact between the asylum-seekers is difficult to prevent. Compulsion did not seem adequate in keeping with the principle of proportionality.

Therefore, the focus was on identifying particularly vulnerable groups of people. Pregnant women and infants were classified as being especially at risk. Therefore, in order to clarify the serological status of this particularly vulnerable group, blood samples were taken from those pregnant women who gave their consent. This measure was also extended the closest family members, provided that they were unable to demonstrate immunity (documented vaccination) to enable families to be accommodated together.

Risk assessment was performed, stratified by cohort within the facility (see Table 3 (Tab. 3)). Since it was observed that the group of Moldavian asylum-seekers in which the index case had occurred had little contact with other ethnic groups, especially with African-born facility resi-dents, it was decided to transfer the African-born pregnant women with their infants and families to a separate facility within the district. The rationale for the segregation of this cohort was, on the one hand, the special hazard to pregnant women and infants in general, and on the other hand, the lower risk of infection for this group. The other pregnant women and infants were isolated within the facility of the index case, because the probability of already existing infection for the latter cohort was rated higher.

The management of relocating individuals, especially mothers discharged postpartum with their newborns and their families, was determined in close consultation with the local public health department for each individual case after careful risk assessment and serological status survey. Until the measures were lifted on 29/08/2018, 35 persons were accommodated in the separate accommodation in the district.

In the period from 02/08/2018 to 04/08/2018, containment vaccinations were again carried out at the facility. This was also carried out at a second facility/group housing where the medical center and the administration is situated. The contacts of the residents between these two facilities were limited to the bare minimum, contacts in the waiting rooms reduced as far as possible, which generally proved difficult. The vaccine offer was continued and intensively advertised in order to motivate as many residents as possible to be vaccinated.

## Results

The outbreak was limited to only two cases. Apart from the index patient, another measles case was reported on 07/08/2018. It was an 18-year-old male patient, also of Moldavan descent. He was identified as the uncle of the index case and had not come to Regensburg with the index case, but had first been transferred to another institution. According to the investigation, however, he had never been to this group housing and had lived elsewhere in Regensburg before he registered at the facility. Clinically, a typical exanthema and catarrhal symptoms were found. Isolation measures were promptly initiated. A laboratory confirmation was received on 10/08/2018. The laboratory constellation showed positive PCR in negative serology.

**Figure 1:**
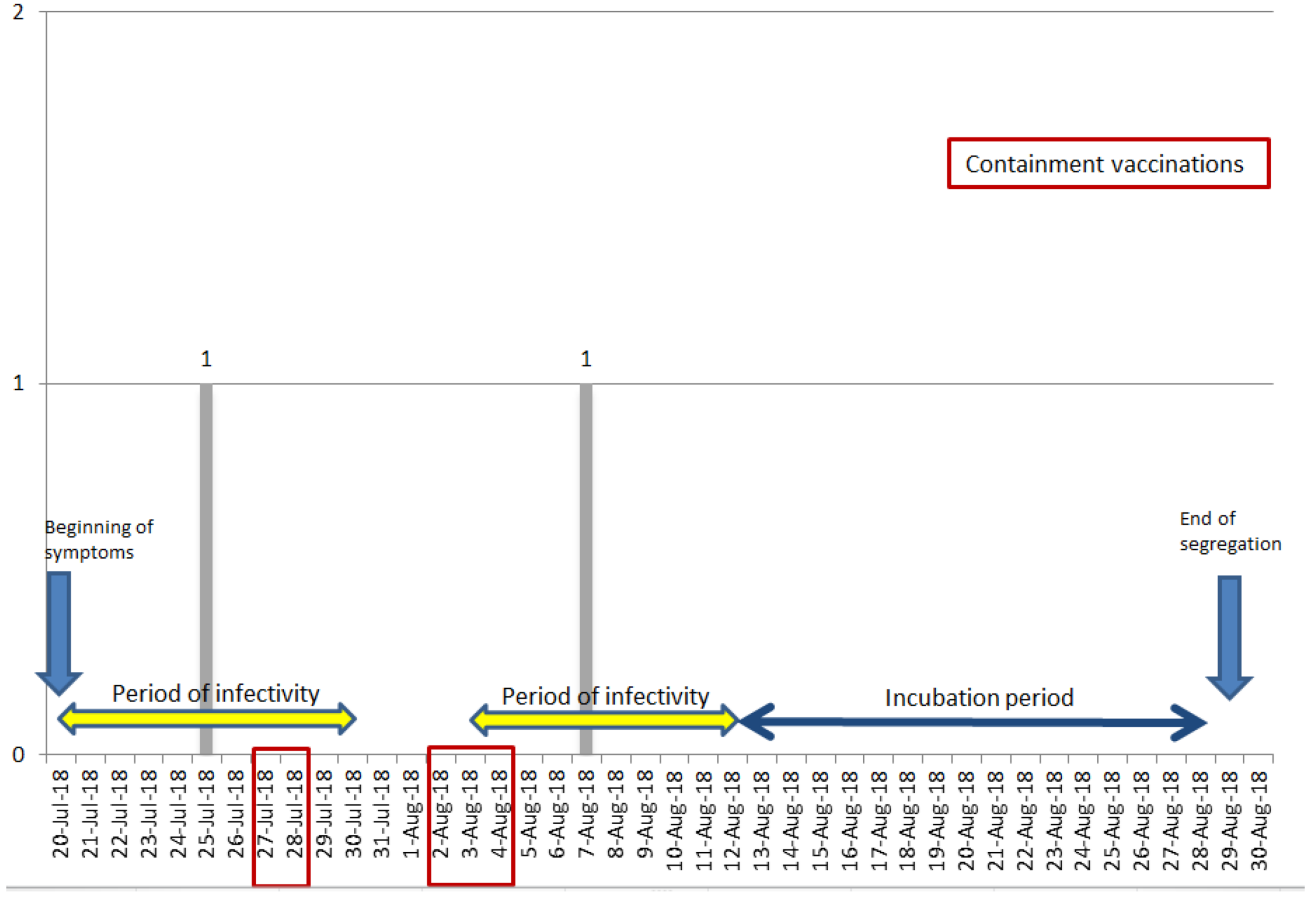
Chronology (epidemic curve)

Based on the occurrence of the rash in the last case reported, the period of infectivity was set to the period 03/08/2018 to 12/08/2018, and the critical period for the incidence of further cases was calculated by the incubation period [1] (data of reported cases and time course s. Table 2 (Tab. 2) and Figure 1 (Fig. 1)). Other cases were not reported during this period, so that on 29/08/2018 all infection control measures could be lifted. Another suspected case, which was initially reported as positive during a serological evaluation, turned out to be immune (see Table 1 (Tab. 1)). No cases of measles were reported from the facility in the neighboring county (Schwandorf), to which persons had been transferred within the days prior to the onset of rash in the index patient until the end of segregation measures in Regensburg.

### Overall outbreak description

The Bavarian Health and Food Safety Authority (LGL) established the following epidemiological background with regard to the source of infection and transmission: The two patients arrived in Germany via Berlin. The 5-year-old index case was, as far as known, housed in Berlin from 21/06/2018 to 11/07/2018 and relocated to Regensburg on 12/07/2018; his uncle stayed in Berlin at the same reception facility from 13/07/2018 to 20/07/2018. In an initial reception facility in Lower Saxony, 3 measles cases were reported in 2 Ukranian families (beginning of exanthemas 24/07/2018 and 27/07/2018, respectively). The affected families had arrived at the initial reception facility on 12/07/2018. One family had been in the Netherlands before arriving, the other family in a reception facility in Berlin. On 08/08/2018 and on 09/08/2018, two more related cases became known.

On 30/08/2018, the Bavarian Health and Food Safety Authority (LGL) was notified by the Public Health Department of Schwandorf (District Oberpfalz) by an event report on a measles case in one facility for asylum-seekers in Schwandorf. It was a 3-year-old girl from Moldova with the onset of exanthema on 29/08/2018. In the course of the investigation, two other patients (brothers) were found in Schwandorf with onset of illness on 24/07/2018 and 15/08/2018. The patients affected had all been previously transferred from the facility in Regensburg. On the basis of these observations, it is highly probably that all 5 cases are in an epidemiological context. Genotyping could not be conducted either in the two patients from Regensburg or the two brothers from Schwandorf. A sample from the 3-year-old girl was genotyped as D8 with the Distinct Sequence ID 5165. 

From Berlin, a case in an unvaccinated girl from Moldova was reported (beginning of symptoms on 25/07/2018 and onset of exanthema on 31.07.2018). In this case, the genotype D8 with the Distinct Sequence ID 5165 was also detected. 

Moreover, three cases from Lower Saxony were reported with the genotype D8-5165. One of the families had been housed in an initial reception facility in Berlin (two cases with the same genotype) before relocation to Lower Saxony. Thus, an epidemiological connection to Berlin seems likely. 

The RKI has reported a total of 15 cases so far containing the sequence variant D8-5165. According to the RKI, D8-5165 derives from the Caucasus (mostly Georgia, also cases in Armenia), other cases were demonstrated in Russia (Moscow) and Poland.

## Discussion

The rates of immunity in residents within the facility at the time of illness onset in the index case are not available and cannot be determined because of missing or incomplete vaccination documents. Based on the general practitioner’s experience on site, however, it can be stated that the willingness to be vaccinated, also depending on the country of origin, varied greatly. This applies equally to both containment and routine vaccinations. Testing for measles-specific antibodies was only performed in pregnant women, mothers of infants and their close relatives in order to allow families to be accommodated together. A routine serostatus determination does not appear appropriate for the high or unknown number of contagion suspects in group housing.

It can be speculated that a high proportion of initially immune individuals contributed to the rapid containment of the outbreak; how high this proportion exactly was cannot be said with certainty. For a purely self-limiting course, however, the vaccination or immunity rate for a disease such as measles with a contagion index near 100, a basic reproduction number R_0_=approx. 16 or a critical vaccination of about 94% [7], respectively, does not seem high enough. The assessment of the overall serological status on site was difficult because it was not a closed cohort sensu stricto, despite the swiftly initiated admission/relocation stop after the announcement of the index case. Relocation and registration were still performed until the index case was confirmed; strict separation between the two mentioned institutions was not possible. This was partly due to the fact that contacts between those accommodated at the two different institutions – especially if they were family members – could not be consistently be prevented, and partly because the administration and the medical care rooms are housed in the unaffected institution.

## Conclusion

The early and consistent implementation of the measures and the close coordination between the responsible persons successfully prevented a larger measles outbreak. The close cooperation was made possible through daily communication between the participants, evaluating the efficacy of the measures in regular meetings, discussing any changes of the strategy and developing a consensus under the primacy of infection control. At every step, the local conditions and numerous structural and organizational implications also had to be taken into account.

From our point of view, decisive factors for the successful management of this outbreak were the early information of all persons involved, the early and repeated execution of containment vaccinations, the consistent segregation of contagion and/or disease suspects, as well as the critical weighing of each individual case in connection with the separate risk-adjusted segregation of particularly vulnerable cohorts (see Table 3 (Tab. 3)).

It was difficult to enforce and maintain the isolation measures in a commensurate manner while simultaneously taking appropriate protective measures especially for the vulnerable group of pregnant women and infants. Identifying and isolating various risk groups proved to be a successful strategy. In this, the local public health department was particularly dependent on the advice of the institution management and local staff, who provided valuable information in this regard.

## Notes

### Acknowledgement

We thank Dorle Matysiak-Klose (Robert-Koch-Institute, Berlin), Julia Bitzegeio, Dirk Werber (Landesamt für Gesundheit und Soziales, Berlin), Konrad Beyrer and Dagmar Ziehm (Niedersächsisches Landesgesundheitsamt, Hanover) for sharing epidemiological information with us.

### Competing interests

The authors declare that they have no competing interests.

## Erratum

Text correction chapter "Results"

## Figures and Tables

**Table 1:**
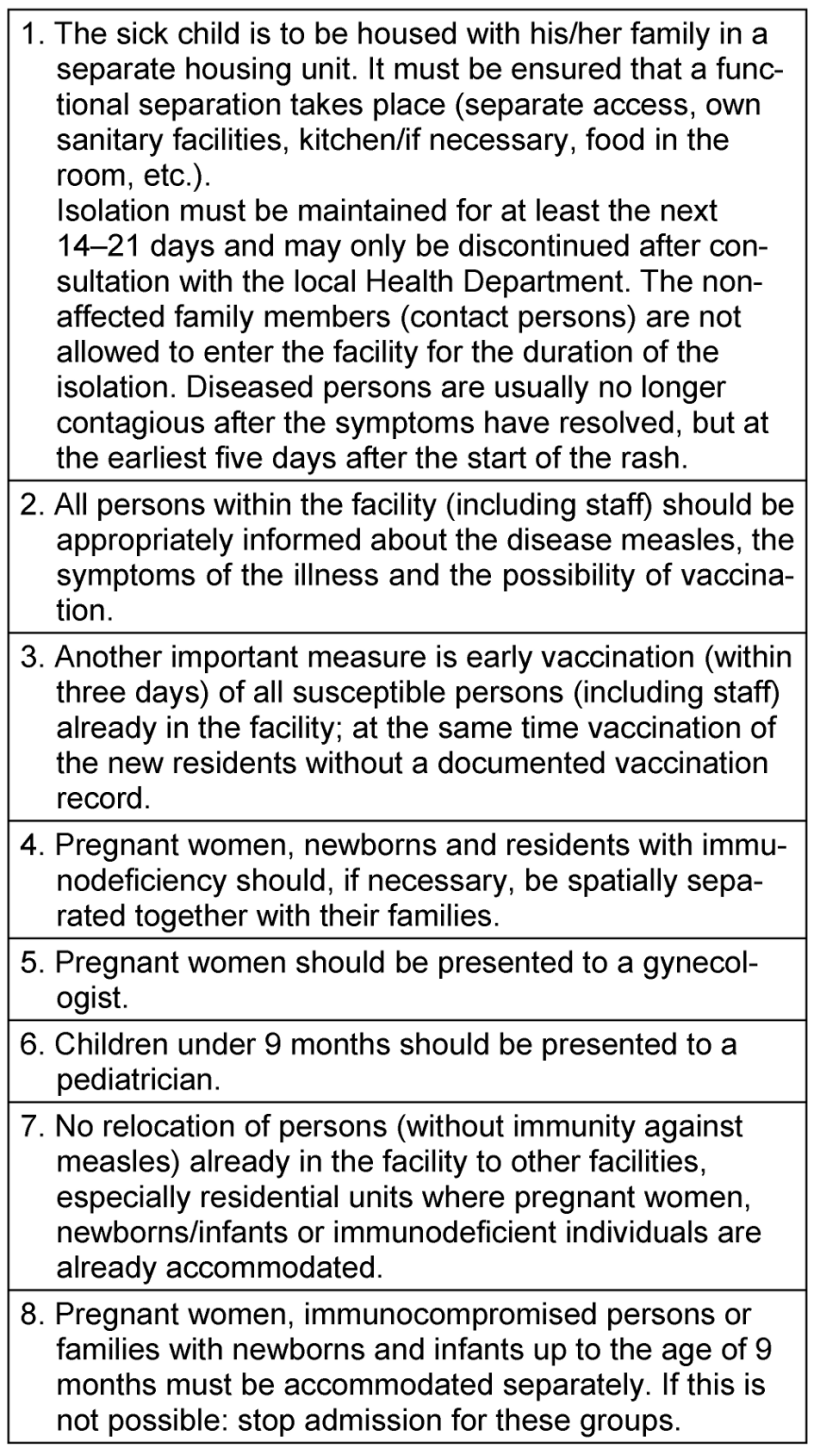
Immediate response to the occurrence of measles in a reception facility, modified as recommended by the LGL ([8], p. 84; [9])

**Table 2:**
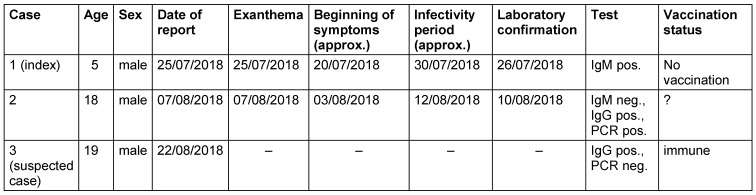
Measles cases from 25/07/2018 to 28/08/2018

**Table 3:**
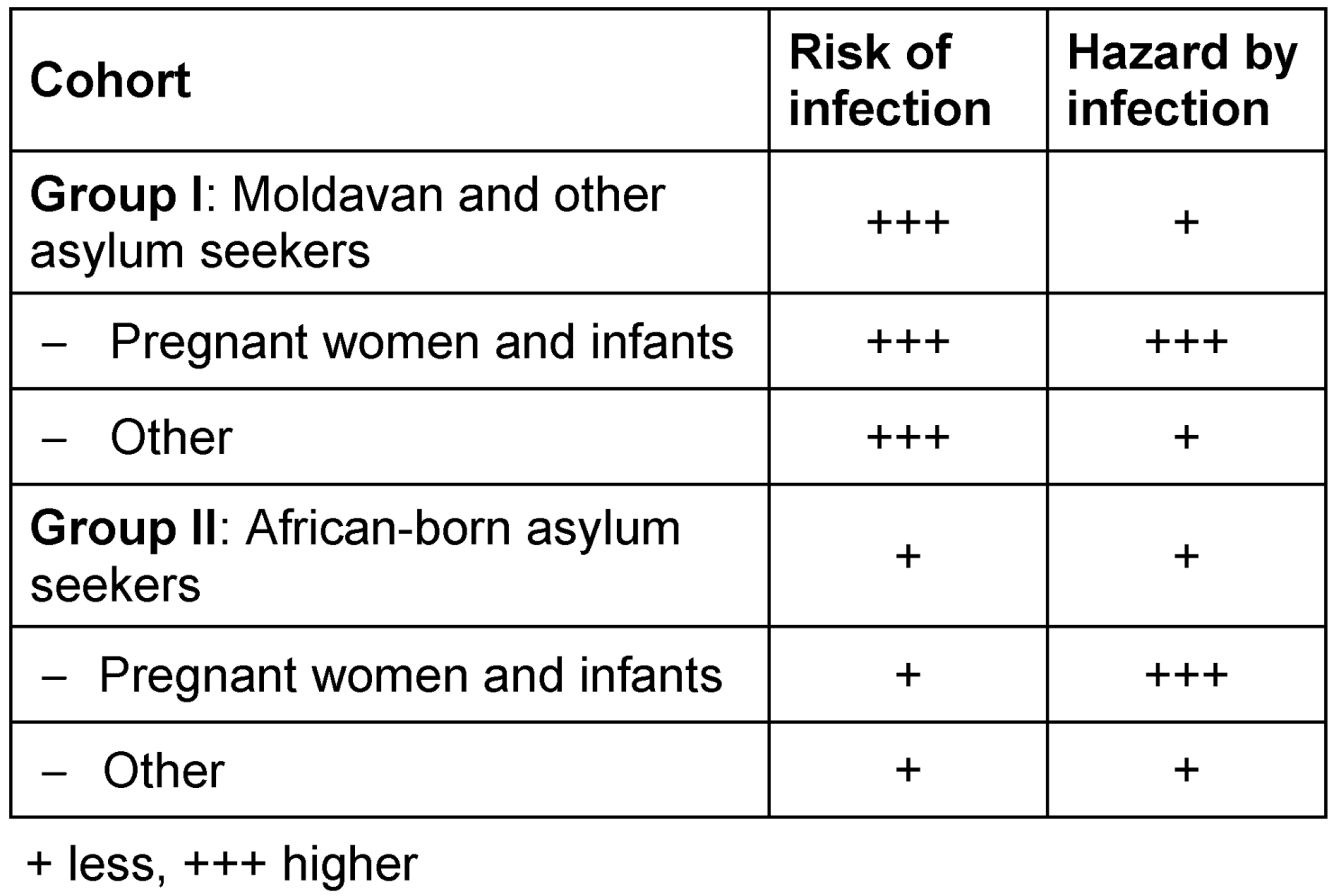
Risk assessment stratified by different cohorts. Rationale for the separate placement of the pregnant women/infants of the African-born group: risk of infection +, hazard by infection +++
